# The Advent of Artificial Intelligence into Cardiac Surgery: A Systematic Review of Our Understanding

**DOI:** 10.21470/1678-9741-2023-0308

**Published:** 2024-12-15

**Authors:** Rahul Bhushan, Vijay Grover

**Affiliations:** 1 Department of Cardiovascular and Thoracic Surgery, All India Institute of Medical Sciences (AIIMS), Patna, India.; 2 Department of Cardiac surgery, Atal Bihari Vajpayee Institute of Medical Sciences (ABVIMS) and Dr Ram Manohar Lohia (RML) Hospital, New Delhi, India.

**Keywords:** Artificial Intelligence, Cardiac Surgical Procedures, Patient Care, Technology

## Abstract

When faced with questions about artificial intelligence (AI), many surgeons respond with scepticism and rejection. However, in the realm of cardiac surgery, it is imperative that we embrace the potential of AI and adopt a proactive mindset. This systematic review utilizes PubMed® to explore the intersection of AI and cardiac surgery since 2017. AI has found applications in various aspects of cardiac surgery, including teaching aids, diagnostics, predictive outcomes, surgical assistance, and expertise. Nevertheless, challenges such as data computation errors, vulnerabilities to malware, and privacy concerns persist. While AI has limitations, its restricted capabilities without cognitive and emotional intelligence should lead us to cautiously and partially embrace this advancing technology to enhance patient care.

## INTRODUCTION

A typical surgeons’ response to every pertinent question related to artificial intelligence (AI) is a shrug of shoulder, evasion, and a roaring, thumping rejection. Few sheepishly submit to changing times and generation as future may befall unforeseen changes to our small world of cardiac surgeons. So, the question lies, how should we respond. Shall we take a step back, recognize a foe, and prepare for war? Shall we embrace with arms wide open a better newer pathway to future patient care. Or shall we stay obnoxious and sit on fence to see how it performs in other fields and then decide accordingly its role in our own territory and domain^[1,2]^.

Our experience with technology hasn’t been great in past. Catheter-based intervention came and snatched and ate up a good chunk of pie from ambit of cardiac surgery. The ever-developing market of transcatheter aortic valve replacement and mitral valve intervention along with angioplasty has been a thorn in our eyes for decades now. Newer generation of cardiac surgeons in India are finally taking up catheter-based intervention with gradual training and learning. We do not wish to fall laggards again when it comes to AI, and a proactive progressive mindset is the need of hour to identify another brewing storm in the field of machine learning and medical care.

## METHODS

This systematic review was performed from database of PubMed® search with keyword of “artificial intelligence, cardiac surgery and medical sciences” and literature was identified from 2017 onwards based on relevance and abstract assessment of studies. We followed the Preferred Reporting Items of Systematic Reviews and Meta-Analyses (or PRISMA) guidelines. A total of 276 papers were reviewed, and 26 were identified and cited for data analysis.

## RESULTS

First, it is important to understand how AI works. It comprises of four subsets in approach, viz, a) machine learning, b) natural language processing, c) artificial neural networks, and d) computer vision^[3]^.

In machine learning, data based on pictures, videos, simulation, live surgery, and algorithms are fed into computers^[4,5]^. This aids the system in identification of structures, formulate ideal surgical steps etc. Based on limitless sources of multiple algorithms, machine learning is highly accurate in recognising subtle patterns and achieve data interpretation beyond human limit from multivariate analysis^[6,7]^.

Via natural language processing, AI ensembles electronic medical data, standard recommendation for surgical practices, identifies trends in postoperative complications and follow-up, and predicts adverse outcomes accordingly^[8,9]^.

The artificial neural network is the game changer in term of superhuman ability to compound the data subset, reads complex pattern, and projects its applied outcome on task execution^[10]^. The “neurons” are expected to work just like human minds in data interpretation and variables calculation^[11]^.

Lastly, computer vision gives mathematical quantifiable data into simplistic image/video form for human interaction and discussion for comprehension. Thus, with supercomputers, fibreoptic data transmission, and advances in ability of models, there is no doubt about efficacy of AI models in whatever application we wish to apply its use into^[12]^. That shall be our first take home message and arguing this fact shall be futile.

Next, one shall ponder upon applicability of this to our field. This is where the challenging decisions comes where we shall be happily welcoming the source and where do we want to put our foot down.

### Artificial Intelligence in Academia

The application begins from literature and teaching process. Few journals have accepted artificial intelligence (AI) as citation source as well as allow use of AI for data accumulation, proofreading, and data review. As cited by a study by Char et al.^[13]^, this can be a welcome step. Our energy, resource, and time shall be much less consumed accepting AI as part of academia rather than rejecting it sitting on the fence with it. The accuracy in data is obviously unparalleled, and a better shift in quality of publication, data review, and interpretation shall be possible by using AI as an assist in our academic front. In residency programmes as well as teaching classes, AI can be aptly put to use for better interactive communication is comprehension of complex cardiac topics to bring in 3D models and put forth concept and competence-based learning to good effect. This was duly pointed out in study conducted by Azari et al.^[14]^ in using AI and its technology for teaching purposes.

In recently published editorial article by Walter J Gomes et al.^[15]^ in BJCVS, the authors discuss boons and pitfalls associated with decision of approval of AI as a language model to be used in scientific writing, as stated by the International Committee of Medical Journal Editors (or ICMJE). This indicates a paradigm shift in accepting AI as language model as a tool in scientific writing aiding to improve manuscript quality by eliminating inaccuracy and errors. However, the authors warn about potential bias and ethical implication. The article underscores a global consensus which encourages academicians to embrace AI to enhance quality publication with due ethics and declaration being made.

Similarly, in an analytical study conducted by Athaluri et al.^[16]^, researchers sought references generated by AI for citation purposes. Out of the 178 references generated by AI, it was found that 69 of these references lacked a Digital Object Identifier (or DOI), and 28 references did not appear in Google search results; instead, they were extracted from books rather than research articles. These findings underscore the pressing need for the thoughtful inclusion of AI within a broader regulatory framework for research publications.

### Artificial Intelligence and Diagnostic Medicine

The role of AI in diagnostic evaluation shall has already proven track record of augmented efficiency and better disease prediction and assessment. AI-based remodelling of computed tomography/magnetic resonance imaging scans, 3D computing, and assessment of ideal surgical approach has already deeply invaded radiology and pathology for better predictive models and is an unavoidable reality. In the paper by Komorowski et al.^[17]^, detection rate of disease and dysfunction varied drastically with and without use of AI. In our field, with clinician dependent varied data analysis, such as estimation of wall motion abnormalities or septal dyskinesia on echocardiogram or evaluation of degree of coronary stenosis with feasibility of transcatheter *vs.* surgical interventions, it is imperative that we embrace use of AI for better predictive and diagnostic models.

### Artificial Intelligence and Risk Assessment

For preoperative risk assessment, multiple models have been postulated as followed upon globally. However, for example, a complex relation between pro-B-type natriuretic peptide levels, elevated creatinine level, and low/high platelet count can be used as predictive analysis for future incidence of infarction in patient planned for coronary revascularization. This complex mathematical unbiased algorithm-based approach is beyond limitations of human mind and it shall be a welcome step in safe planning and execution of cardiac surgery^[18,19]^. In the study by Ana et al.^[20]^, an intraoperative better prediction of management of bleeding or hypotension or an enhanced need for ionotropic support base on visual parameters of cardiac contractility with its algorithm-based correlation with pulmonary arterial pressure, central venous line pressure, and variation in height of spike of dicrotic notch etc can be achieved based on simple mathematical calculations. Thus, AI can prove to be an indispensable tool for anaesthesiologists as well as perfusionists in better execution of cardiac surgery.

### Artifical Intelligence in Surgery and Postoperative Care

For decision on use of AI in actual surgery, the debate gets heated. From proponents of complete human elimination in surgery to believers in human supremacy in real time-based event response and sensory response to touch, feel of heart and vessel, and multiple parameters for visual and sensory perception, the debate is endless^[21,22,23]^. Not caving into this debate and staying on fence, we, for the time being, shall be most ideally accepting AI as an assist and a tool intraoperatively for recommendation, guidance, and related predictive outcome models for discussion-based approach and surgical outcome in an ideal scenario^[24,25,26,27]^. In the article authored by Qin Pei et al.^[28]^, which details the utilization of AI in comprehensive patient management for cases of lung cancer, the study revealed a profound impact of AI in aiding patient management. AI proved to be immensely valuable not only in imaging but also in providing accurate diagnoses with better delineation of tumour-free margins and establishing resectability criteria. Furthermore, it facilitated surgeons in proposing the ideal surgical approach and strategy during procedures. The seamless integration of AI with robotic technology may represent the next step in the future of applied AI, further enhancing surgical accuracy and outcomes.

Again, in the postoperative period, a consensual input from AI on ideal management approach, medications, fluid management, and ionotropic support shall be way forward^[29,30,31]^. In a study conducted by Sohvi et al.^[32]^, the utilization of AI in tailoring individualized drug dosages and strategies for diverse population phenotypes was explored. The findings demonstrated that employing machine learning and algorithm-based approaches for personalized prescriptions proved to be more effective in determining optimal drug usage, dosages, and treatment durations.

## DISCUSSION

In the realm of AI, there exist notable limitations that challenge its role as a universal solution. These limitations encompass occasional errors in data computation, variations, and processing, vulnerabilities to malware, viruses, and predatory programs, as well as deficiencies in data safeguards and patient privacy documentation. Addressing these issues necessitates careful consideration and human oversight.

Recent research by Lucinado et al.^[33]^ has shed light on the pitfalls associated with the use of AI in research publications. This work underscores the ongoing debate surrounding AI’s role in scientific literature, touching upon concerns related to authorship attribution, responsibility, and the potential for misuse in disseminating misinformation during critical events like pandemics. These considerations raise fundamental questions about the need for regulations and the ethical application of AI within the scientific community.

Furthermore, Geoffrey M. Currie’s analysis delves into the significant drawbacks of employing AI-based language models as publication tools. Currie emphasizes the inherent risks, including errors and the potential for information fabrication, associated with AI usage. Consequently, a compelling case emerges for the establishment of regulatory guidelines and a governing body within the publication domain. Such oversight can effectively address malpractices while also promoting language editing and the constructive utilization of AI to elevate research quality^[34,35]^.

### Limitations

The limitation of this study is ever changing sphere of artificial intelligence with limited available literature.

## CONCLUSION

The absence of cognitive and emotional intelligence underscores the limitations of AI. Embracing its potential with caution and partial acceptance is vital. A complete rejection of AI is impractical, and by recognizing its benefits and limitations, we position ourselves to navigate this transformative landscape effectively.
